# THINKING ABOUT TIME INFLUENCES EMOTIONAL EXPERIENCE DIFFERENTLY IN OLDER AND YOUNGER ADULTS

**DOI:** 10.1093/geroni/igad104.1835

**Published:** 2023-12-21

**Authors:** Enna Chen, Claire Growney, Laura Carstensen

**Affiliations:** Stanford University, Stanford, California, United States; Stanford University, Stanford, California, United States; Stanford, Stanford, California, United States

## Abstract

According to socioemotional selectivity theory (SST), as future time horizons shorten with age, people come to prioritize present-oriented, emotionally meaningful goals. SST posits that present orientation contributes to emotional well-being. Using experience sampling, the present study examined (1) age differences in momentary time orientations, (2) associations between momentary time orientations and emotional well-being, and (3) age moderation of these associations. A sample of 190 people, aged 18 to 94 years, participated in an ecological momentary assessment study where they simultaneously reported time orientations and experienced emotions at five randomly selected times every day for seven days. . We found that age was not associated with time orientation. However, on occasions when people thought about the past, they experienced less positive affect and more negative affect. On occasions when people thought about the present, they experienced more positive affect. All of these effects were weaker for older as compared to younger participants. However, the association between thinking about the future and positive or negative affect did not vary by age. Findings suggest that younger adults, even more than older adults, benefit emotionally from focusing on the present.

